# Effects of a large-scale social media advertising campaign on holiday travel and COVID-19 infections: a cluster randomized controlled trial

**DOI:** 10.1038/s41591-021-01487-3

**Published:** 2021-08-19

**Authors:** Emily Breza, Fatima Cody Stanford, Marcella Alsan, Burak Alsan, Abhijit Banerjee, Arun G. Chandrasekhar, Sarah Eichmeyer, Traci Glushko, Paul Goldsmith-Pinkham, Kelly Holland, Emily Hoppe, Mohit Karnani, Sarah Liegl, Tristan Loisel, Lucy Ogbu-Nwobodo, Benjamin A. Olken, Carlos Torres, Pierre-Luc Vautrey, Erica T. Warner, Susan Wootton, Esther Duflo

**Affiliations:** 1 Harvard University, Department of Economics, Cambridge, MA USA; 2 Massachusetts General Hospital, Department of Medicine, Neuroendocrine Unit, Department of Pediatrics, Endocrinology, Boston, MA USA; 3 Harvard Medical School, Boston, MA USA; 4 Harvard Kennedy School of Government, Cambridge, MA USA; 5 Online Care Group, Boston, MA USA; 6 Massachusetts Institute of Technology, Department of Economics, Cambridge, MA USA; 7 Stanford University, Department of Economics, Stanford, CA USA; 8 Ludwig Maximilian University of Munich, Department of Economics, Munich, Germany; 9 Bozeman Health Deaconess Hospital, Bozeman, MT USA; 10 Yale University, School of Management, New Haven, CT USA; 11 Lynn Community Health Center, Lynn, MA USA; 12 Johns Hopkins University, School of Nursing, Baltimore, MD USA; 13 St. Anthony North Family Medicine, Westminster, CO USA; 14 Paris School of Economics, Paris, France; 15 Massachusetts General Hospital, Department of Psychiatry, Boston, MA USA; 16 McLean Hospital, Department of Psychiatry, Belmont, MA USA; 17 Massachusetts General Hospital for Children, Department of Pediatrics, General Pediatrics, Boston, MA USA; 18 Massachusetts General Hospital, Department of Medicine, Clinical and Translational Epidemiology Unit, Mongan Institute, Boston, MA USA; 19 McGovern Medical School at The University of Texas Health Science Center at Houston, Houston, TX USA

**Keywords:** Lifestyle modification, Health care economics, Randomized controlled trials

## Abstract

During the Coronavirus Disease 2019 (COVID-19) epidemic, many health professionals used social media to promote preventative health behaviors. We conducted a randomized controlled trial of the effect of a Facebook advertising campaign consisting of short videos recorded by doctors and nurses to encourage users to stay at home for the Thanksgiving and Christmas holidays (NCT04644328 and AEARCTR-0006821). We randomly assigned counties to high intensity (*n* = 410 (386) at Thanksgiving (Christmas)) or low intensity (*n* = 410 (381)). The intervention was delivered to a large fraction of Facebook subscribers in 75% and 25% of randomly assigned zip codes in high- and low-intensity counties, respectively. In total, 6,998 (6,716) zip codes were included, and 11,954,109 (23,302,290) users were reached at Thanksgiving (Christmas). The first two primary outcomes were holiday travel and fraction leaving home, both measured using mobile phone location data of Facebook users. Average distance traveled in high-intensity counties decreased by −0.993 percentage points (95% confidence interval (CI): –1.616, −0.371; *P* = 0.002) for the 3 days before each holiday compared to low-intensity counties. The fraction of people who left home on the holiday was not significantly affected (adjusted difference: 0.030; 95% CI: −0.361, 0.420; *P* = 0.881). The third primary outcome was COVID-19 infections recorded at the zip code level in the 2-week period starting 5 days after the holiday. Infections declined by 3.5% (adjusted 95% CI: −6.2%, −0.7%; *P* = 0.013) in intervention compared to control zip codes. Social media messages recorded by health professionals before the winter holidays in the United States led to a significant reduction in holiday travel and subsequent COVID-19 infections.

## Abstract (web summary)

A new cluster randomized controlled trial conducted in 13 American states demonstrates that a social media advertising campaign using videos of healthcare professionals to encourage users to stay at home over the holiday season was effective in reducing travel and subsequent spread of COVID-19.

## Main

Nurses and physicians are among the most trusted experts in the United States^1–3^. However, it is unknown if these healthcare professionals can influence behavior at scale by spreading public health messages using social media. During the COVID-19 crisis, many healthcare professionals in the United States used social media to spread public health messages^3^. For example, the Kaiser Family Foundation sponsored a large project where doctors recorded videos to provide explanations about COVID-19 vaccination and dispel doubts^1^. Because individual adoption of preventative behavior, from mask wearing and staying at home to vaccination, is key to the control of the current and future pandemics, it is important to know whether this communication strategy is effective. In previous work, we used online experiments to show that video messages, recorded by a diverse group of doctors, affect the knowledge and behaviors of individuals and that these effects seem to be strong regardless of race, education or political leanings^4,5^. However, there is no systematic evaluation of similar messages when distributed as part of large-scale public health campaigns. Furthermore, given the large sample required for such experiments, it has not been possible to test the effect of public health campaigns on COVID-19 infection; thus, the clinical implications of our preliminary findings are unclear.

In this study, we sought to estimate whether short video messages recorded by nurses and doctors and sent on a massive scale as part of a social media advertising campaign could affect human behavior and COVID-19 infections at the zip code (cluster) level. In November 2020, the number of COVID-19 cases was rapidly increasing across the United States. Owing to concerns that holiday travel would lead to a surge in the epidemic, the Centers for Disease Control and Prevention recommended that people stay home for the holidays^6^. In this context, we ran a large clustered randomized controlled trial with users of Facebook, a platform that covers approximately 70% of American adults^7^. Before Thanksgiving and Christmas, physicians and nurses (F.C.S., M.A., B.A., T.G., K.H., E.H., S.L., L.O-N., C.T., E.T.W. and S.W.) recorded 20-s videos on their smartphones to encourage users to stay home for the holidays. Facebook subscribers in randomly selected zip codes in 820 counties in 13 states received these videos as sponsored content (ads; Methods). Over 11 million people received at least one ad before Thanksgiving (35% of users in the targeted regions), and over 23 million people received at least one video before Christmas (66% of users in targeted regions). On average, each user we reached received 2.6 videos at Thanksgiving and 3.5 at Christmas, usually with a different doctor represented. The message was always the same at Thanksgiving and had small variations at Christmas (see Methods for full scripts and videos). The purpose of this study was to identify whether these short videos would influence population-level holiday travel in the targeted regions and, in turn, cause a decline in COVID-19 cases after the holidays.

## Results

### Trial population

**Fig. 1:**
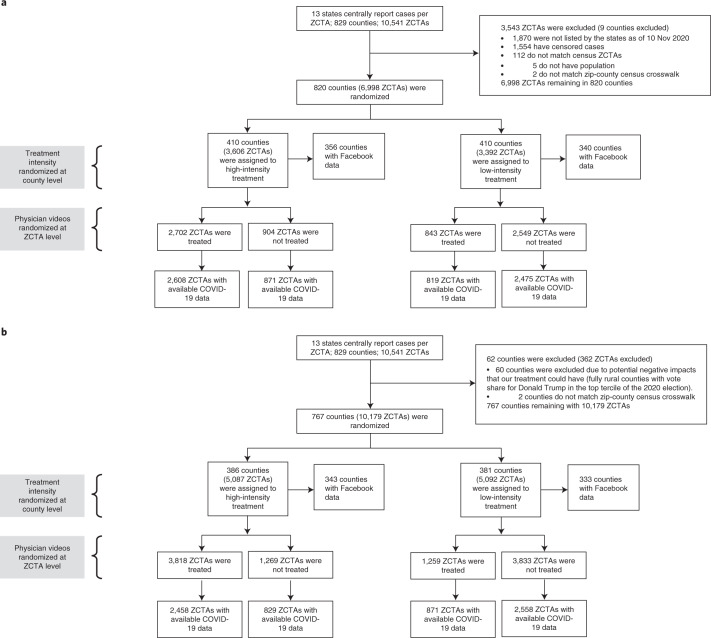
Trial design. **a**, CONSORT diagram for the Thanksgiving campaign. **b**, CONSORT diagram for the Christmas campaign. ZCTA denotes zip code tabulation area (zip codes).

**Extended Data Fig. 1:**
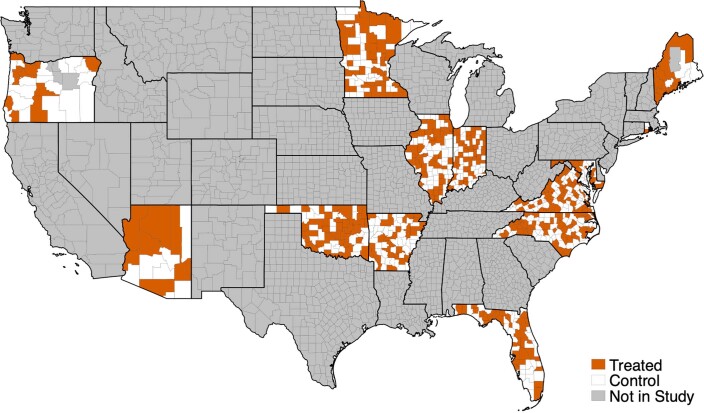
Randomized counties (Thanksgiving campaign). Source data

**Extended Data Fig. 2:**
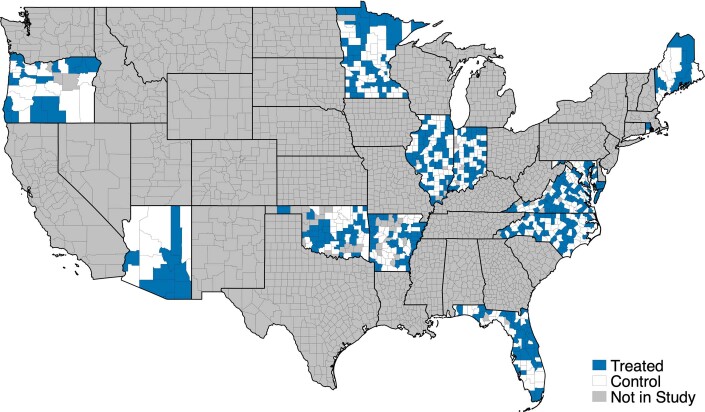
Randomized counties (Christmas campaign). Source data

The CONSORT diagram (Fig. 1) describes the factorial design and the allocation of clusters to each arm. Before the Thanksgiving campaign, we selected 13 states where weekly COVID-19 case count data were available at the zip code level (see maps in Extended Data Figs. 1 and 2) and selected counties within these states where these data were available. Counties are administrative units of the United States, with an average population of about 100,000, whereas zip codes are postal codes and are smaller than counties. There are more than 40,000 zip codes and approximately 3,100 counties in the United States.

Of the 8,671 potentially eligible zip codes in the 13 states in the study, 1,554 were removed before the Thanksgiving campaign because of missing COVID-19 infection data, and 119 were removed because they could not be matched to county-level census data, yielding a sample of 6,998 zip codes in 820 counties. Before the Christmas campaign, 60 fully rural counties in the top tercile of votes for Donald Trump in the 2020 election were removed from the study. This was done out of caution and to avoid adverse effects. Given the growing polarization between the presidential election and the inauguration, and considering a small number of extremely negative and politically charged comments posted during the Thanksgiving campaign, the research team was concerned that the messaging campaign might have adverse unintended effects in very rural, heavily Republican-leaning counties.

At Thanksgiving, 410 counties were allocated to the high-intensity group, including 356 with Facebook mobility data (Facebook does not release county-level data when the number of users is below 300). In those counties, 2,608 zip codes were treated and had COVID-19 infection data, and 871 counties were treated and had COVID-19 infection data. In total, 410 counties were allocated to the low-intensity group (including 343 with Facebook mobility data). In those counties, 819 zip codes were treated and had COVID-19 data, and 2,475 zip codes were controls and had COVID-19 data. Because the counties excluded from the Christmas campaign were very small and, as a result, are often excluded from the public release mobility data set we used, we lost only 20 counties with county-level mobility data between Christmas and Thanksgiving. Thus, at Christmas, 386 counties were allocated to the high-intensity group (including 340 with mobility data), and 381 counties were allocated to the low-intensity group (including 333 with mobility data). In high-intensity counties, 2,485 zip codes were treated and had COVID-19 infection data, and 829 counties were controls and had COVID-19 infection data. In low-intensity counties, 871 zip codes were treated and had COVID-19 infection data, and 2,558 counties were controls and had COVID-19 infection data. The realized sample size was similar to the original sample size and did not lead to substantial loss in power.

Summary statistics on the sample that was randomized are shown in Table 1 (Extended Data Figs. 1 and 2 in the Supplementary Appendix show their localization on the map). As is generally true in the United States, most counties lean Republican^8^). Counties had, on average, 36% Democrats and 62% Republicans (based on election share in 2020), and 46% of zip codes were classified as urban. On 13 November 2020, the distance traveled was 8.73% lower than during the benchmark month in the Facebook Movement Range data of February 2020. In the Christmas sample, it was 8.89% lower. In both samples, 82.4% of people left their homes on 13 November 2020.

**Table 1:** Summary statistics

	Thanksgiving sample			Christmas sample		
	Sample	High-intensity counties	Low-intensity counties	Sample	High-intensity counties	Low-intensity counties
Number of counties	820	410	410	767	386	381
Movement, mean (s.d.)						
Baseline movement metric	−8.73 (6.77)	−8.58 (7.10)	−8.88 (6.42)	−8.89 (6.72)	−8.69 (6.88)	−9.09 (6.56)
Baseline leave home	82.41 (2.47)	82.33 (2.42)	82.49 (2.53)	82.42 (2.41)	82.40 (2.43)	82.44 (2.40)
Missing baseline Facebook outcomes	0.15 (0.36)	0.13 (0.34)	0.17 (0.38)	0.12 (0.32)	0.11 (0.32)	0.13 (0.33)
COVID-19, mean (s.d.)						
Baseline fortnightly cases	590.30 (2,297.94)	683.90 (3,032.94)	496.70 (1,165.17)	626.84 (2,371.71)	654.77 (3,067.53)	598.54 (1,343.02)
Baseline fortnightly deaths	5.07 (17.63)	5.51 (22.35)	4.64 (11.08)	5.38 (18.19)	5.70 (23.07)	5.07 (11.29)
Demographic, mean (s.d.)						
Share urban	0.46 (0.34)	0.47 (0.34)	0.44 (0.34)	0.49 (0.33)	0.48 (0.33)	0.50 (0.33)
Share Democrats	0.36 (0.15)	0.36 (0.15)	0.35 (0.15)	0.37 (0.15)	0.37 (0.15)	0.37 (0.15)
Share Republicans	0.62 (0.15)	0.62 (0.16)	0.63 (0.15)	0.61 (0.15)	0.61 (0.15)	0.61 (0.15)
Population in 2019	112,654 (317,672)	122,491 (349,501)	102,818 (282,369)	119,811 (327,266)	116,787 (344,511)	122,875 (309,239)

Summary statistics on baseline variables, for both Thanksgiving and Christmas samples. Baseline = 13 November 2020.

### Effects of the campaign on the mobility of Facebook users

**Fig. 2:**
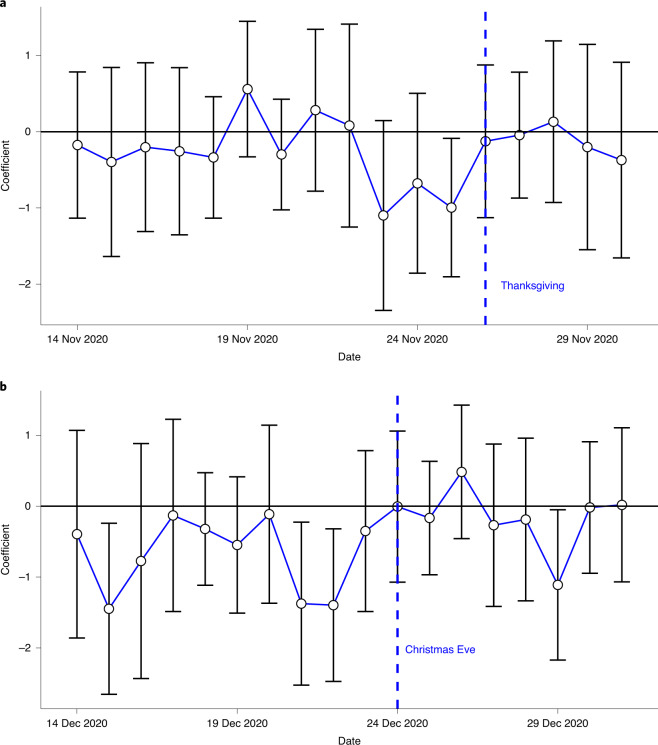
Day-by-day difference between high- and low-intensity counties on distance traveled relative to February 2020. **a**, Thanksgiving campaign (*n* = 696 counties). **b**, Christmas campaign (*n* = 677 counties). Day-by-day estimation of the regression Eq. (1). Each dot represents the difference in distance traveled relative to February 2020 between high- and low-intensity counties on the specified day. The whiskers are the 95% CIs. Source data

**Extended Data Fig. 3:**
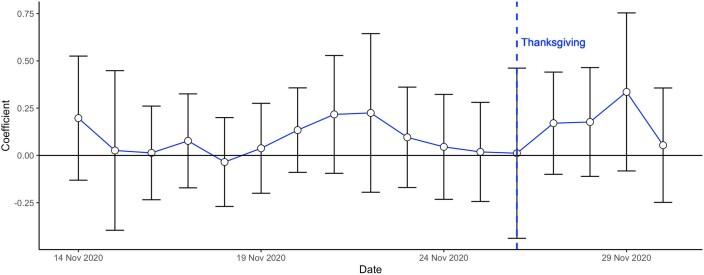
Day by day difference between high and low intensity counties on Share Ever Left Home (Thanksgiving campaign). Day by day estimation of the regression Eq. (1). Each dot represents the difference in share of people leaving home between high and low intensity counties on the specified day. The whiskers are the 95% confidence intervals. n = 696 counties. Source data

**Extended Data Fig. 4:**
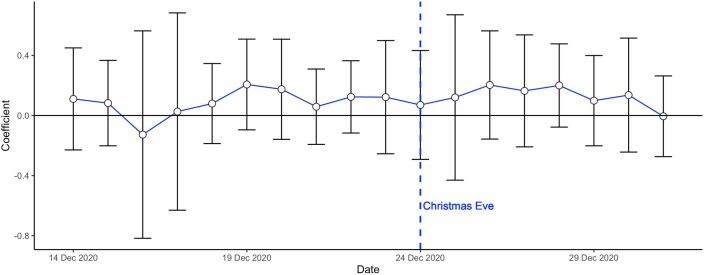
Day by day difference between high and low intensity counties on Share Ever Left Home (Christmas campaign). Day by day estimation of the regression Eq. (1). Each dot represents the difference in share of people leaving home between high and low intensity counties on the specified day. The whiskers are the 95% confidence intervals. *n* = 677 counties. Source data

Figure 2 shows day-by-day regression estimates of Eq. (1) (Methods), along with 95% CI. For the Thanksgiving campaign, the distance traveled away from the morning location declined on 22, 23 and 24 November (3 days before the Thanksgiving holiday on 25 November) in high-intensity counties relative to low-intensity counties. For the Christmas campaign, it was lower on 21 and 22 December in high-intensity counties relative to low-intensity counties. Pooling both campaigns together, the distance traveled during the 3 days before each holiday was 4.384% lower than in February 2020 in high-intensity counties and 3.597% lower in low-intensity counties (Table 2). The adjusted difference was 0.993 percentage points (95% CI: −1.616, −0.371; *P* = 0.002). The effects were similar at Thanksgiving (adjusted difference: −0.924 percentage points; 95% CI: −1.785, −0.063; *P* = 0.035) and Christmas (adjusted difference: −1.041 percentage points; 95% CI: −1.847, −0.235; *P* = 0.011). The intervention had no effect on the share of people leaving home on the day of the holiday (Table 2 and Extended Data Figs. 3 and 4). On average, 72.33% of people left home (specifically, a 600-m area centered around their home) on the day of the holiday in high-intensity counties, and 72.39% of people left home in low-intensity counties (adjusted difference: 0.030; 95% CI: −0.361, 0.420; *P* = 0.881). These results are robust to adding control variables chosen by machine learning from a large set of county-level covariates (Supplementary Table 10). Supplementary Table 11 shows that, furthermore, the effects were found at all quantiles of the mobility distribution and were not driven by tail events (Supplementary Table 11).

**Table 2:** Effect of treatment on movement outcomes

			Mean (95% CI)		OLS model			Number of days × counties
Campaign	Outcome	Period	High-intensity county	Low-intensity county	High-intensity county (95% CI)	*P* value	RI *P* value	
Both campaigns	Distance traveled	From day − 3 to day − 1	−4.384 (−4.973,−3.796)	−3.603 (−4.254, −2.952)	−0.993 (−1.616, −0.371)	0.002	0.002	4,059
Both campaigns	Share ever left home	Thanksgiving (26 November) or Christmas (24–25 December)	72.326 (72.012, 72.639)	72.381 (72.092, 72.670)	0.030 (−0.361, 0.420)	0.881	0.911	2,017
Thanksgiving	Distance traveled	From day − 3 to day − 1	−6.082 (−6.822, −5.341)	−5.320 (−6.113, −4.527)	−0.924 (−1.785, −0.063)	0.035	0.030	2,072
Thanksgiving	Share ever left home	Thanksgiving (26 November)	71.308 (70.885, 71.731)	71.468 (71.071, 71.866)	0.012 (−0.438, 0.461)	0.959	0.966	689
Christmas	Distance traveled	From day − 3 to day − 1	−2.603 (−3.279, −1.927)	−1.823 (−2.588, −1.057)	−1.041 (−1.847, −0.235)	0.011	0.008	1,987
Christmas	Share ever left home	Christmas (24–25 December)	72.859 (72.507, 73.210)	72.852 (72.520, 73.185)	0.095 (−0.289, 0.479)	0.629	0.580	1,328

The control and treatment means at the county level and different periods, in addition to the estimate of the treatment coefficient in Eq. (1). Standard errors are clustered at the county level. 95% CIs are reported in parentheses. *P* values are based on a two-sided test. RI *P* values are computed using randomization inference, accounting for the two-stage design.

### Effect of the campaign on COVID-19 cases

Table 3 shows that the campaigns were followed by a drop in COVID-19 cases in treated zip codes relative to control zip codes for the 2-week period beginning 5 days after the holiday. The adjusted difference in the inverse hyperbolic sine of COVID-19 cases was 0.035 (adjusted 95% CI: −0.062, −0.007; *P* = 0.013), which can be interpreted as a 3.5% reduction in COVID-19 cases (Methods). The effects were slightly smaller and not significant at the 5% level at Thanksgiving (adjusted difference: −0.027; 95% CI: –0.059, 0.005; *P* = 0.097) compared to the effects at Christmas (adjusted difference: −0.042; 95% CI: −0.073, −0.012; *P* = 0.007). These results are robust to alternative ways to treat zeros (Supplementary Tables 14, 15 and 16). The quantile regressions in Supplementary Tables 12 and 13 show that the effects were found at all levels of the distribution and were not driven by tail levels (because there are relatively few zeros, only the lowest quantiles are affected by the way zeros are handled).

**Table 3:** Treatment effect on COVID-19 cases at the zip code level

			Mean (CI 95%)		OLS model			Number of zip codes
Campaign	Period	County treatment	Treatment	Control	Treatment (CI 95%)	*P* value	RI *P* value	
Both campaigns	December/1–14 January	All	4.350 (4.302, 4.398)	4.370 (4.323, 4.417)	−0.035 (−0.062, −0.007)	0.013	0.005	13,489
Both campaigns	December/ 1–14 January	Low intensity	4.359 (4.273, 4.445)	4.358 (4.305, 4.411)	−0.032 (−0.067, 0.004)	0.080	0.062	6,723
Both campaigns	December/1–14 January	High intensity	4.347 (4.295, 4.399)	4.407 (4.325, 4.489)	−0.039 (−0.075, −0.003)	0.033	0.021	6,766
Thanksgiving	1–14 December	All	4.333 (4.278, 4.388)	4.298 (4.243, 4.353)	−0.027 (−0.059, 0.005)	0.097	0.108	6,773
Thanksgiving	1–14 December	Low intensity	4.284 (4.170, 4.399)	4.256 (4.192, 4.320)	−0.015 (−0.063, 0.033)	0.535	0.498	3,294
Thanksgiving	1–14 December	High intensity	4.348 (4.285, 4.411)	4.418 (4.313, 4.523)	−0.039 (−0.082, 0.004)	0.078	0.096	3,479
Christmas	1–14 January	All	4.368 (4.310, 4.425)	4.442 (4.385, 4.499)	−0.042 (−0.073, −0.012)	0.007	0.010	6,716
Christmas	1–14 January	Low intensity	4.429 (4.312, 4.547)	4.456 (4.391, 4.522)	−0.048 (−0.091, −0.006)	0.025	0.043	3,429
Christmas	1–14 January	High intensity	4.346 (4.280, 4.412)	4.396 (4.281, 4.510)	−0.036 (−0.080, 0.008)	0.108	0.111	3,287

The control and treatment means at the zip code level, in addition to the estimate of the treatment coefficient in Eq. (2). The outcome is the inverse hyperbolic sine of the fortnightly cases, during a period that starts 5–7 days after the event (Thanksgiving or Christmas). 95% CIs are reported in parentheses. Standard errors are clustered at the zip code level. *P* values are based on a two-sided test. RI *P* values are computed by randomization inference, accounting for the two-stage design.

**Fig. 3:**
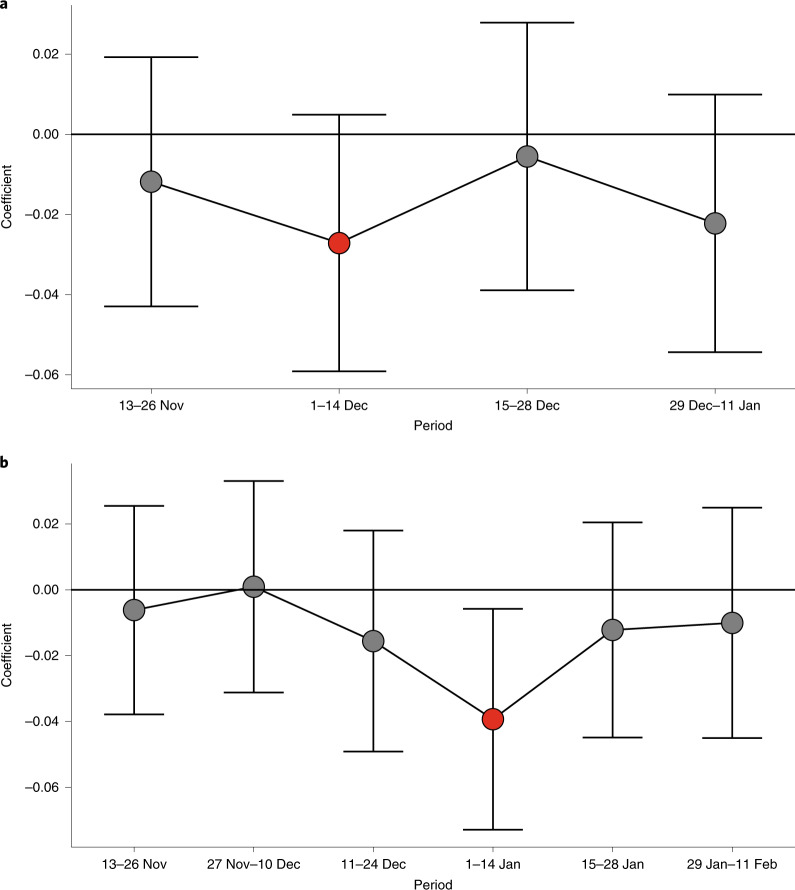
Difference between treated and control zip codes in inverse hyperbolic sine numbers of COVID-19 infection by 2-week periods. **a**, Thanksgiving campaign (*n* = 6,773 zip codes). **b**, Christmas campaign (*n* = 6,716 zip codes). Estimation of the regression Eq. (2) for each fortnight. Each dot represents the differences in the inverse hyperbolic sine of COVID-19 cases between treated and control zip codes for the given 2-week period. The whiskers are the 95% CIs. The red dot denotes the period that is directly affected by each campaign. Source data

To provide evidence that these differences were due to the campaign and not to any pre-existing differences, we used estimating Eq. (2) for several 2-week periods (omitting the 5 days after each holiday) at Thanksgiving (Fig. 3a) and Christmas (Fig. 3b). There was no significant difference between intervention and comparison zip codes in any pre-intervention period, making it very unlikely that the difference in COVID-19 cases was due to random chance. In addition, the effect seems to be concentrated in the 2 weeks immediately after the holiday, especially for Christmas, suggesting that the effects were not persistent.

### Treatment effect heterogeneity

We tested for several pre-specified dimensions of heterogeneity of the effect of the campaign on mobility and COVID-19 infection: baseline COVID-19 infections, urban versus rural counties and majority Republican counties versus majority Democratic counties (Supplementary Tables 1–8).

We found no significant difference in the effects of the campaign either on mobility or COVID-19 cases between Republican and Democratic counties or between rural and urban counties. We also did not find that the interaction between political leaning and urban designation was significant (Supplementary Tables 7 and 8). The effects on COVID-19 infections are smaller in magnitude in counties with high infections at baseline (Supplementary Table 2).

We include one non-pre-specified heterogeneity analysis. We compared the effects on COVID-19 cases after Thanksgiving in zip codes located in counties that were excluded at Christmas versus those that were included (this could not be done for mobility because only 20 of those counties had mobility data). We found no significant difference (Supplementary Table 9).

### Harms and unintended effects

We did not expect any harm to individuals from this study, unless a severe backlash effect had led to an adverse effect on travel, leading to an unintended effect on COVID-19 cases. We did not find evidence for such heterogeneity, suggesting that no harmful or unintended effects occurred.

## Discussion

There was widespread concern before the Thanksgiving and Christmas holidays that heavy travel and mixing households from different regions would lead to an increase in COVID-19 infections. Indeed, households did travel more around the holidays, even with mobility staying lower than the levels in February 2020.

In counties where a larger proportion of zip codes were randomly assigned to a high-coverage Facebook ad campaign in which clinicians encouraged people to stay home before the Thanksgiving and Christmas holidays, Facebook users reduced the distance they traveled in the 3 days before the holidays. They were no more likely to stay at home on the day of the holiday. However, the clinical importance of this second finding is unclear, because we do not know what they did while not at home. Participants could have been outdoors or celebrating with other families locally. Furthermore, local celebrations might have different epidemiological implications than associating with households farther away.

The sign and magnitude of the effects are consistent with other evidence that social gatherings have contributed to the spread of COVID-19. In counties in the top decile of COVID-19 prevalence, the presence of a birthday in the last 2 weeks (presumably associated with more social gatherings) was associated with a 31% increase in the chance of a new COVID-19 infection, with lower effects in other deciles^9^. We found an average reduction of 3.5% in the number of new COVID-19 infections from our campaign that aimed to reduce holiday travel, but the campaign did not lead people to stay home. The results are also consistent with observational estimates of the effect of non-pharmaceutical interventions in Europe, which found very large reductions in *R* due to full lockdowns and much smaller, but still detectable, effects of other non-pharmaceutical interventions (self-isolation, bans on public events, school closures and encouragement of social distancing)^10^.

A potential concern before the campaign was that, in a polarized environment, a campaign such as this one could be effective in some communities and backfire in others (which is why we excluded 60 heavily Republican rural counties). But the effects did not seem to depend on county characteristics, including political leanings, both at Thanksgiving and at Christmas. Although present, the effects could have been somewhat muted by the exclusion of the 60 heavily Republican-leaning counties. Nevertheless, our findings accord with previous research that found that individuals are responsive to physician-delivered messages, regardless of political affiliation^5^, and that increases in COVID-19 infection after birthdays exist both in Democratic- and Republican-leaning counties^9^.

We found a significant effect on new COVID-19 infections reported by health authorities 5–19 days after the ad campaigns. These effects might be underestimated because the treatment and control zip codes are very close to each other, and the reductions in infection in treatment zip codes might also have led to a decrease in infections in neighboring places. In the presence of spillovers, the degree of attenuation might also be different in high-intensity versus low-intensity counties. For example, the indirect spillover effect on control zip codes might be larger in high-intensity counties. There is evidence consistent with this hypothesis at Christmas but not at Thanksgiving. We plan to conduct a more detailed spillover analysis in follow-up work, using the Social Connectedness Index constructed from the interactions on the Facebook platform^11,12^.

This study had several limitations, in particular regarding generalizability. First, the study was conducted with Facebook subscribers, and mobility data were collected for Facebook users only. Although Facebook has an extensive reach (70% of Americans are users), this remains just one type of media. Second, it was an ad campaign. The messages might have been more or less effective if they had been relayed by celebrities or locally known figures, as we have tested in other work^13,14^. Third, we tested one kind of message, recorded by clinicians on smartphones. The results could be different if variables such as message content, identity of the messenger, length of message, production value of the videos or name recognition of the originating organization were varied. Fourth, our results speak to the effects of messaging campaigns lasting only 1–2 weeks before the holidays. The effects of longer-run, repeated messaging might be smaller if recipients become fatigued or lose interest in the content.

Despite these limitations, our findings provide evidence that clinicians can be an effective channel to communicate lifesaving information at scale through social media. This is a new role that physicians and nurses embraced during the COVID-19 crisis, and we demonstrate that this is another way in which they can prevent illness and save lives. These findings also demonstrate, in a clustered randomized control trial, the effects of travel reduction, which is a key non-clinical intervention whose effect had not been previously evaluated in a randomized controlled trial. The findings suggest directions for future work, particularly investigating if similar messages could be effective in encouraging COVID-19 vaccine uptake.

## Methods

### Trial oversight

The design was approved by the institutional review board (IRB) of the Massachusetts Institute of Technology (MIT) with Massachusetts General Hospital, Yale and Harvard ceding authority to the MIT IRB. Messages were produced by the research team and approved to run (without modification) after going through Facebook’s internal policy review to ensure compliance with policies. The study was registered on ClinicalTrials.gov (NCT04644328) and the American Economic Association registry for randomized social experiments (AEARCTR-0006821).

The IRB protocol and relevant amendments are available online (along with the data and code). Procedures that apply to this specific project are highlighted. There was no deviation from the IRB protocol. Further details on the design were registered in a statistical analysis plan and on the ClinicalTrials.gov registration. The analysis in this paper focuses only on the primary outcomes registered on ClinicalTrials.gov. There was just one deviation from the pre-registration in ClinicalTrials.gov: we initially planned to construct zip code-level mobility outcomes from fine-grained data, but, owing to privacy concerns, those data are not available for this purpose. Because the publicly available mobility data are at the county level, we used only county-level mobility data (which we had always planned to use).

The sample, the specific unit of randomization, the randomization methods and planned analyses were pre-registered before the Thanksgiving campaign in a publicly available statistical plan (10.1257/rct.6821-1.0). We followed the analysis plan for the primary outcomes outlined on 25 November for both the Christmas and Thanksgiving campaigns in the 13 states, with two deviations. First, we were not able to obtain publicly available zip code-level mobility outcome from fine-grained data, so we used only county-level data on mobility. Second, we had not specified a functional form for the number of COVID-19 cases. We specify one below.

In this paper, we focus on the direct effects of the intervention in the 13 original experimental states on the primary outcomes specified in the ClinicalTrials.gov registration. Although the statistical analysis plan also discusses indirect spillover effects, an extension of the campaign in ten new states 3 days before Thanksgiving and various supplementary analyses, these are left for follow-on work.

### Intervention

Messages encouraging viewers to stay home for the holidays were recorded on smartphones by six physicians before Thanksgiving and nine physicians and nurses before Christmas who varied in age, gender, race and ethnicity (see the Supplementary Material for examples of videos).

For Thanksgiving, video script was:

‘This Thanksgiving, the best way to show your love is to stay home. If you do visit, wear a mask at all times. I’m [Title/ NAME] from [INSTITUTION], and I’m urging you: don’t risk spreading COVID. Stay safe, stay home’.

For Christmas, the video script was

‘We are nurses and doctors, and, this year, as hard as it is, we are staying home for the holidays. I am doing this because [REASON]. I am Dr [DOCTOR’S NAME] from [DOCTOR’S INSTITUTION], and I am urging you: don’t spread COVID-19. Stay stafe, stay home’.

The reasons given were one of the following:

‘I have seen too much suffering in my hospital’, ‘It is the safest way to celebrate’, ‘I love you, Mom and Dad’, ‘There is light at the end of the tunnel, and we just need to hang in there a little bit longer’.

The videos were then disseminated as sponsored content to Facebook users from a page created for the project. The videos and the Facebook page are available on the project website: https://www.povertyactionlab.org/project/covid19psa.

#### Details about Facebook ad campaigns

We disseminated the messages using a Facebook ad campaign that was managed by AdGlow, our marketing partner. On the Facebook advertising platform, there are many ways to structure a campaign. We selected a ‘reach’ objective, which attempts to maximize the number of Facebook users seeing the ads, along with the number of times each user sees the ad, over a daily horizon or the lifetime of the campaign, given the campaign budget. The Thanksgiving campaign had a daily ‘reach’ objective, whereas the Christmas campaign had a lifetime ‘reach’ objective. Facebook uses an algorithm to implement the campaign objective. (More information is available at https://www.facebook.com/business/help/218841515201583?id=816009278750214.)

An important element of the algorithm is the Facebook Ads Auction. All active ad campaigns define a target audience. For both of our campaigns, the target audience consisted of all Facebook users in the specified zip codes. Every time there is an opportunity to show an ad to a user, there might be many active campaigns targeting that type of individual. An auction is used to determine the cost of the ad and which ad is shown to the user at that time, and the auction winner is the advertiser with the highest total value. Total value is a combination of three factors: the bid of each advertiser; the estimated action rate (whether the user engages with the ad in the desired way); and ad quality, which is measured by Facebook and reflects feedback from previous viewers and assessments of so-called ‘low-quality attributes’. By defining total value as more than simply the advertiser’s bid, ads that are estimated to create more user engagement or that are of higher quality can beat ads with higher bids in the auction. In our study, the Facebook ad campaign algorithm and Ads Auction led to the delivery of campaign materials to 11,954,108 users at Thanksgiving and 23,302,290 users at Christmas. (More information about the Facebook Ads Auction is available at https://www.facebook.com/business/help/430291176997542?id=561906377587030.)

### Trial design, eligibility, randomization and recruitment

Eligibility for the trial and the cluster randomization strategy were determined by data availability and power considerations. Movement range data computed by Facebook are publicly available at the county level. COVID-19-level data are available at the zip code level in the states where we conducted this experiment.

To ensure that we would have adequate power for both the mobility and the COVID-19 outcomes with publicly available data, we randomized at both the county and zip code levels to generate experimental variation for each set of outcomes.

The CONSORT diagram (Fig. 1) describes the factorial design and the allocation of clusters to each arm.

Before the Thanksgiving campaign, we selected 13 states where weekly COVID-19 case count data were available at the zip code level (see the maps in Extended Data Figs. 1 and 2) and selected counties within these states where these data were available.

The research team randomly allocated counties to be ‘high-intensity’ (H) or ‘low-intensity’ (L) with probability ½ each. In H counties (blocking by county), the research team randomized zip codes into intervention with probability ¾ and control with probability ¼. In L counties (blocking by county), zip codes were randomized into intervention with probability ¼ and control with probability ¾. Randomization was performed by the research team with Stata before each of the two interventions. This two-stage randomization ensures that there is randomized variation in the intensity of the campaign at both the county level and the zip code level. The county-level randomized variation allows us to measure the effect of a high-intensity versus low-intensity campaign on mobility using publicly available data. The zip code-level randomized variation allows for a direct comparison of treated and control zip codes. Because there was no individual randomization and recruitment, concealment was not necessary, and consent was not sought. No one in the team was blinded to the randomization.

The lists of zip codes for each intervention were then provided to our marketing partner AdGlow, which managed the advertising campaigns on Facebook. Within the treated zip codes, AdGlow ran ads to allocate the sponsored video content to users, aiming to reach the largest number of people given the advertising budget. In each ad, users were shown one video message from a set of 14 videos at Thanksgiving and 20 at Christmas. The video messages were pushed directly into users’ Facebook feeds (approximately three times per user on average), and users were then free to watch, share, react to or entirely ignore the content. The intervention was targeted to the cluster. We did not recruit individuals for the study and did not use individual-level data. At Thanksgiving, 30,780,409 ads were pushed to 11,954,109 users between 14 and 29 November, and, at Christmas, 80,773,006 ads were pushed to 23,302,290 users between 17 and 31 December.

AdGlow provided us with overall engagement figures. Each time a user had an opportunity to view a campaign message, 12.3% watched at least 3 s of the video at Thanksgiving and 12.9% watched at least 3 s of the video at Christmas, whereas 1.7% watched at least 15 s at Thanksgiving and 1.4% watched at least 15 s at Christmas. Our engagement rates of 12–13% (measured as the total of clicks, 3-s views, shares, likes and comments divided by total impressions) were well above industry standard benchmarks for Facebook ads, which are 1%-2%, and Facebook video posts, which are 6%^15,16^.

We determined that a sample of 820 counties would provide 80% power to detect effect sizes of 0.2 standard deviations for county-level outcomes, comparing intervention (H) versus control (L). Because our analysis was based on outcomes after taking county-level averages, no intra-class correlation assumption is needed. For outcomes with zip code-level data, using intra-class correlations of 0.2 (0.475) assuming clusters of equal size, a sample of 6,998 zip codes would provide 80% power to detect effect sizes of 0.057 (0.072) standard deviations (using zip code-level data). For the zip code-level outcomes, power calculations were conducted via simulation. In both cases, our calculations were based on a 5% type I error rate in two-sided tests.

### Outcomes

Our primary outcomes are county-level mobility (distanced traveled and leave home metric) and zip code-level COVID-19 infections reported to state health authorities, which we regularly retrieved from state websites beginning on 12 November 2020 and ending on 11 February 2021.

### County-level mobility data

Our mobility outcomes come from the publicly available Facebook Movement Range dataset, which can be downloaded at https://data.humdata.org/dataset/movement-range-maps. The data are constructed from location information collected by Facebook from users who have opted into location history sharing and are aggregated to the county level. The publicly released data are subjected to a differential privacy framework to maintain the privacy of individual Facebook users. First, regions with fewer than 300 users on a given date are omitted from the dataset (so we lose small counties). Second, random noise is added during the construction of each metric to limit the risk of being able to identify individual users^17,18^. We used mobility data from 14 November to 31 December 2020.

We used both the Change in Movement metric and the Stay Put metric in our analysis. Both are calculated daily and cover the period from 20:00 to 19:59 local time. Both metrics are based off of changes in locations across level-16 Bing tiles, which each represent an area of approximately 600 m × 600 m.

Change in Movement is a measure of how many tiles the average Facebook user starting in a given county travels through during the day. More specifically, the variable is constructed for each county on each day following five steps: (1) the number of tiles visited is calculated for each user and is top-coded at 200; (2) the total number of tiles visited by all users in that county-day observation is calculated by summing over the top-coded tiles measure; (3) random noise is added to the total tiles measure following a Laplace distribution with parameters selected to satisfy Facebook’s differential privacy targets; (4) the noisy total tiles variable is scaled by Facebook users observed in the data to generate an average for that day in each county; and (5) the average movement measure is scaled by an average baseline measurement for the county taken on the same day of the week between 2 and 29 February 2020.

Stay Put is calculated as the fraction of observed users in a given county who do not leave a single level-16 Bing tile for the whole day. Specifically, in constructing the public version of this metric, five steps are followed: (1) a binary indicator is calculated for each user based on whether they remained in a single level-16 Bing tile for the entire day; (2) the total number of users in each county staying put is generated; and steps (3)−(5) from the Change in Movement calculation are followed. When we use the Stay Put metric in our analysis, we instead create Leave Home = 1 − Stay Put, so that larger values indicate more movement.

The Facebook Movement Range data are described in further detail at https://research.fb.com/blog/2020/06/protecting-privacy-in-facebook-mobility-data-during-the-covid-19-response/.

The mobility data describe the behavior throughout the day for people who were in each county *that morning*. Because the campaign was targeted based on home location, we can only capture its effect on travel *away* from home, not back home. Thus, we define holiday travel as travel preceding each holiday. We focus on the 3 days before each holiday to capture the busiest periods of travel. We also present day-by-day effects graphically over a longer horizon. The *stay put metric* is the share of people who stay within a small geographical area (a ‘Bing tile’ of 600 m × 600 m) in which they started the day. We used it to compute the *leave home* variable as = 1 − *stay put* on the day of the holiday (Thanksgiving Day, Christmas Eve and Christmas Day).

### COVID-19 cases

The third primary outcome that we studied is the number of new COVID-19 cases per fortnight, calculated from the cumulative case counts that we manually retrieved from county or state webpages and cleaned. Our primary outcome is the number of new COVID-19 cases detected in each zip code during the fortnight that starts 5 days after each holiday: given the incubation period of 5 d, this is the one 2-week period where we should see an effect. We use COVID-19 infection data collected from 12 November 2020 to 11 February 2021. We stopped collecting that data because we had enough post-intervention data to measure immediate and longer-run effects.

The COVID-19 data were retrieved twice a week from each state’s public health website. The data are reported by hospitals or labs to the centralized state-wide health department, which publishes the data that we collected and used. Most states report positive cases based on polymerase chain reaction tests, but some (AZ, IL and MN) combine confirmed with probable cases. Data for most of the zip codes were updated on the websites at only a weekly frequency. The data were retrieved manually and then cleaned and organized.

States reported the cumulative cases in each zip code. Cases are assigned to a zip code based on the address of the person who tested positive.

Some zip codes were not listed on the states’ websites. (We observed around 8,000 unique zip codes before dropping the censored ones, whereas the total zip code count for these 13 states is a bit over 10,000). There are multiple reasons for this, the most common being aggregation of small zip codes into larger ones (there were other situations, like suppressing Tribal zip codes, or simply suppressing small zip codes instead of aggregating them), and the data were censored for zip codes with low case counts.

We cleaned and appended all the data that we collected, totaling 6,998 unique zip codes with unsuppressed, non-censored data.

A list of the websites from which the data were retrieved appears here:

AZ: https://www.azdhs.gov/covid19/data/index.php

AR: https://achi.net/covid19/

FL: https://experience.arcgis.com/experience/96dd742462124fa0b38ddedb9b25e429

IL: https://www.dph.illinois.gov/covid19/covid19-statistics

IN: https://hub.mph.in.gov/dataset?q=COVID

ME: https://www.maine.gov/dhhs/mecdc/infectious-disease/epi/airborne/coronavirus/data.shtml

MD: https://coronavirus.maryland.gov/datasets/mdcovid19-master-zip-code-cases/data

MN: https://www.health.state.mn.us/diseases/coronavirus/stats/index.html

NC: https://covid19.ncdhhs.gov/dashboard

OK: https://looker-dashboards.ok.gov/embed/dashboards/80

OR: https://govstatus.egov.com/OR-OHA-COVID-19

RI: https://ri-department-of-health-covid-19-data-rihealth.hub.arcgis.com/

VA: https://www.vdh.virginia.gov/coronavirus/covid-19-data-insights/

### Statistical analysis

The analysis was performed by original assigned group (intention to treat), following Eqs. 1 and 2 in the statistical plan.

- Effect on mobility (county level)
  At the county level, the analysis compares the ‘high-intensity’ counties to the ‘low-intensity’ counties. Because, on average, only 75% of the zip codes in high-intensity counties received the intervention, and 25% in low-intensity counties received the intervention, this is ‘an intention-to-treat’ specification, which is a lower absolute bound of the effect of treatment.
  For any day or set of days, the coefficient of interest is *β*_1_ in the ordinary least squares (OLS) regression:
  1 $$y_{it} = \beta _0 + \beta _1\rm{High}_i + \beta _2y_{i0} + {{{\boldsymbol{X}}}}_{{{\boldsymbol{i}}}}\beta _3 + \varepsilon _{it}$$
  where *y*_*it*_ is the outcome of interest on day *t*, and *y*_*i*0_ is its baseline value. This regression is estimated for both campaigns together and for each separately. Standard errors are adjusted for heteroskedasticity and clustering at the county level^19^. CIs are constructed using a *t*-distribution. We also provide randomization inference (RI) *P* values^20^. As pre-specified in the analysis plan, we present a regression controlling for state fixed effects and a set of county-level outcomes chosen via machine learning^21^ as well as quantile regressions.
- Effect on number of COVID-19 cases (zip code level)

To measure the effect on COVID-19 cases reported in each zip code, we ran the regression:

2 $$\begin{array}{l}{\mathrm{Asinh}}\left( {\mathrm{fortnightly}}\;{\mathrm {COVID}}_{\it{it}} \right) =\\\quad {\beta _{0}} + {\beta _{1}}{\mathrm {Treated}}_{\it{i}} + {\beta _2}\log \left( {\rm{cumulative}}\;{\mathrm {COVID}}_{\it{i0}} \right) + {\beta _3^{\boldsymbol{T}}}{{{\bf{stratum}}}}_{{{\boldsymbol{i}}}} + \varepsilon _{it}\end{array}$$

where ‘fortnightly COVID_*it*_’ is the number of new cases of COVID-19 detected in the fortnight beginning 5 days after each holiday (for primary outcome results), and ‘Treated_*i*_’ is a dummy that indicates that zip code *i* was a treated zip code. We also investigated robustness by estimating the same regression for other fortnights (Fig. 3).

The hyperbolic sine transformation is appropriate when the data are approximately log-normal for higher values, but a small number of observations have zero cases^22,23^.

Specifically, the hyperbolic sine function is given by $sinh\left( x \right) = \frac{{e^x - e^{ - x}}}{2}$, and the inverse hyperbolic sine function is given by $asinh\left( x \right) = ln\left( {x + \sqrt {x^2 + 1} } \right)$ .

We chose to transform the fortnightly cases with this function, because it has the property of being equivalent to *x* close to 0 and equivalent to *ln* (*x*) when *x* → +∞: $asinh\left( x \right)\sim _{x \to 0} + x,\;asinh\left( x \right)\sim _{x \to + \infty }ln\left( x \right)$. It behaves like a logarithm for most our our observations, except that there is no singularity at 0.

The coefficient on ‘Treated’ can be interpreted as a proportional change. In the Supplementary Material, we explored robustness to other commonly used ways to handle zeros (Supplementary Tables 14, 15 and 16).

Regression (2) is estimated for both campaigns pooled and for the Thanksgiving campaign and the Christmas campaign separately, with county fixed effects (the randomization strata). Standard errors are adjusted for heteroskedasticity and clustering at the zip code level (15). CIs are constructed using a *t*-distribution, and we also computed *P* values with RI (16). We estimated the effect of treatment overall and separately in the two strata (high-intensity and low-intensity counties).

In the Supplementary Material, we also explored heterogeneity of effects by prior COVID-19 circulation and demographic variables and present quantile regressions.

Analyses were performed using R version 4.0.3, including the following packages (versions): stats (4.0.3), tidyverse (1.3.0), estimatr (0.28.0), readr (1.4.0), dplyr (1.0.5), lubridate (1.7.10), hdm (0.3.1), car (3.0.10), MASS (7.3.53), sandwich (3.0.0), foreign (0.8.80), readstata13 (0.9.2), readxl (1.3.1) and quantreg (5.75).

### Reporting Summary

Further information on research design is available in the Nature Research Reporting Summary linked to this article.

## Online content

Any methods, additional references, Nature Research reporting summaries, source data, extended data, supplementary information, acknowledgements, peer review information; details of author contributions and competing interests; and statements of data and code availability are available at 10.1038/s41591-021-01487-3.

## Supplementary information

Supplementary Information Supplementary Tables 1–16

Reporting Summary

Supplementary Video 1

Supplementary Video 2

## Data Availability

All Facebook data used in the analysis are publicly available to anyone at https://dataforgood.fb.com/docs/covid19/, and no restricted data were used to generate the primary outcomes. All data are shared in a public registry (Harvard MIT data archive). The data are freely accessible at 10.7910/DVN/4EK4KX. Source data are provided with this paper.

## Source data

Source Data Fig. 2 Coefficients, lower bounds and upper bounds
Source Data Fig. 3 Coefficients, lower bounds and upper bounds
Source Data Extended Data Fig. 1 County treatment status
Source Data Extended Data Fig. 2 County treatment status
Source Data Extended Data Fig. 3 Coefficients, lower bounds and upper bounds
Source Data Extended Data Fig. 4 Coefficients, lower bounds and upper bounds

## Code availability

All codes are available in a public registry (Harvard MIT data archive). The code is freely accessible at 10.7910/DVN/4EK4KX. Source data are provided with this paper.
