# Supplementary material for: Effects of a large-scale social media advertising campaign on holiday travel and COVID-19 infections: a cluster randomized controlled trial
Source: Nat Med. 2021 Aug 19;27(9):1622–8. doi: 10.1038/s41591-021-01487-3 (PMC8440209; doi:10.1038/s41591-021-01487-3)
Supplement: Supplementary file 1 — Supplementary Tables 1–16 [file 41591_2021_1487_MOESM1_ESM.pdf]

nature portfolio

https://doi.org/10.1038/s41591-021-01487-3

Supplementary information

Effects of a large-scale social media advertising campaign on holiday travel and COVID-19 infections: a cluster randomized controlled trial

In the format provided by the authors and unedited

Supplementary Appendix

Table of Contents

Supplemental Table 1. Analyses of Mobility Outcomes by Baseline Covid-19 Cases*

Supplemental Table 2. Analyses of Covid-19 Outcome by Baseline Covid-19 Cases *

Supplemental Table 3. Analyses of Mobility Outcomes by Party Majority*

Supplemental Table 4. Analyses of Covid Outcome by Party Majority*

Supplemental Table 5. Analyses of Mobility Outcomes: Urban vs Rural*

Supplemental Table 6. Analyses of Covid Outcome: Urban vs Rural *

Supplemental Table 7. Analyses of Mobility Outcomes by Republican Majority x Urban Majority*

Supplemental Table 8. Analyses of Covid Outcome by Republican Majority x Urban Majority*

Supplemental Table 9. Analyses of Covid Outcome by Excluded counties *

Supplemental Table 10. Effect of Intervention on Movement Outcomes, with Double Post Lasso Control Variables*

Supplemental Table 11. Effect of Intervention on Mobility: quantile regression*

Supplemental Table 12. Effect of Intervention on Covid-19 Outcome (Asinh(Fortnightly Cases)): quantile regression*

Supplemental Table 13. Effect of Intervention on Covid-19 Outcome (Log(Fortnightly Cases +1)): quantile regression*

Supplemental Table 14. Effect of Intervention on Covid-19 Outcome (both campaigns), robustness to function form

Supplemental Table 15. Effect of Intervention on Covid-19 Outcome (Thanksgiving campaign), robustness to functional form

Supplemental Table 16. Effect of Intervention on Covid-19 Outcome (Christmas campaign), robustness to functional form

List of Investigators

Emily Breza, Ph.D.,[¶] Fatima Cody Stanford, M.D. M.P.H, M.P.A.,M.B.A.,[‡,§,*] Marcella Alsan, M.D. Ph.D.,[†,*] Burak Alsan, M.D.,[#] Abhijit Banerjee, Ph.D.,[||] Arun G. Chandrasekhar, Ph.D.,[**] Sarah Eichmeyer, Ph.D., [***] Traci Glushko, M.S.,[##] Paul Goldsmith-Pinkham, Ph.D.,[††] Kelly Holland, M.D.,[‡‡‡] Emily Hoppe, M.S., [§§] Mohit Karnani, M.Sc. [||], Sarah Liegl, M.D., [|||] Tristan Loisel, M.Sc. [†††], Lucy Ogbu-Nwobodo, M.D. M.S. M.A.S., [§,‡‡,¶¶] Benjamin A. Olken Ph.D.,[||] Carlos Torres, M.D., [§,§§§] Pierre-Luc Vautrey, M.Sc. [||], Erica Warner, Sc.D., M.P,H., ,[‡,§,*] Susan Wootton, M.D., [¶¶¶] Esther Duflo, Ph.D.[||]

Affiliations:

[¶] Harvard University, Department of Economics, Cambridge, MA
[†] Harvard Kennedy School of Government, Cambridge, MA
[#] Online Care Group, Boston, MA
[‡] Massachusetts General Hospital, Department of Medicine- Neuroendocrine Unit, Department of Pediatrics- Endocrinology, Boston, MA
[§] Harvard Medical School, Boston, MA
[||] Massachusetts Institute of Technology, Department of Economics, Cambridge, MA
[**] Stanford University, Department of Economics, Stanford, CA
[***] Ludwig Maximilian University of Munich, Department of Economics, Munich, Germany
[##] Bozeman Health Deaconess Hospital, Bozeman, MT
[††] Yale University, New Haven, CT
[‡‡‡] Lynn Community Health Center, Lynn MA
[§§] Johns Hopkins University, School of Nursing, Baltimore, MD
[|||] St. Anthony North Family Medicine, Westminster, CO
[‡‡] Massachusetts General Hospital, Department of Psychiatry, Boston, MA
[§§§] Massachusetts General Hospital for Children, Department of Pediatrics- General Pediatrics, Boston, MA
[¶¶] McLean Hospital, Department of Psychiatry, Belmont, MA

[†††] Paris School of Economics, Paris, France
[¶¶¶] McGovern Medical School at The University of Texas Health Science Center at Houston, Houston, TX

Supplemental Table 1. Analyses of Mobility Outcomes by Baseline Covid-19 Cases*

			OLS model						Number of days * counties
Campaign	Outcome	Period	High county High baseline	p-value	High county	p-value	High baseline	p-value	
Both campaigns	Distance Traveled	from d-3 to d-1	0.811 (-0.579,2.202)	0.253	-1.484 (-2.736,-0.231)	0.020	-0.518 (-1.727,0.690)	0.401	4059
Both campaigns	Share Ever Left Home	Thanksgiving (Nov 26)/ Christmas (Dec 24-25)	-0.471 (-1.309,0.368)	0.271	0.325 (-0.380,1.029)	0.367	0.695 (0.128,1.263)	0.016	2017
Thanksgiving	Distance Traveled	from d-3 to d-1	1.509 (-0.369,3.387)	0.115	-1.813 (-3.479,-0.146)	0.033	-0.744 (-2.210,0.722)	0.320	2072
Thanksgiving	Share Ever Left Home	Thanksgiving (Nov 26)	0.082 (-0.889,1.053)	0.869	-0.052 (-0.889,0.784)	0.903	0.404 (-0.255,1.064)	0.230	689
Christmas	Distance Traveled	from d-3 to d-1	0.738 (-1.110,2.586)	0.434	-1.518 (-3.179,0.142)	0.073	-0.853 (-2.297,0.590)	0.247	1987
Christmas	Share Ever Left Home	Christmas (Dec 24-25)	-0.123 (-0.993,0.746)	0.781	0.181 (-0.580,0.943)	0.640	0.300 (-0.323,0.922)	0.345	1328

*This Table gives the estimates of equation (1) coefficients (here, an interaction with High Baseline was added to the equation) .

Standard errors are clustered at the county level. 95% CI are reported in parentheses. High Baseline is defined as: Cumulative Covid-19 county cases per capita at baseline above median.

Supplemental Table 2. Analyses of Covid-19 Outcome by Baseline Covid-19 Cases *

			OLS model						Number of zip codes
Campaign	Period	County treatment	Treated x High baseline	p-value	Treated	p-value	High baseline	p-value	
Both campaigns	dec/jan 01-14	All	0.047 (-0.003,0.096)	0.065	-0.058 (-0.102,-0.014)	0.009	0.311 (0.259,0.363)	0.000	13489
Both campaigns	dec/jan 01-14	Low Intensity	0.059 (-0.015,0.133)	0.119	-0.061 (-0.122,0.001)	0.054	0.165 (0.105,0.225)	0.000	6723
Both campaigns	dec/jan 01-14	High Intensity	0.047 (-0.027,0.121)	0.214	-0.064 (-0.127,-0.001)	0.048	0.240 (0.159,0.321)	0.000	6766
Thanksgiving	dec 01-14	All	0.039 (-0.022,0.101)	0.208	-0.047 (-0.100,0.006)	0.082	0.095 (0.031,0.159)	0.004	6773
Thanksgiving	dec 01-14	Low Intensity	0.035 (-0.065,0.135)	0.496	-0.033 (-0.115,0.049)	0.434	0.075 (-0.010,0.159)	0.082	3294
Thanksgiving	dec 01-14	High Intensity	0.048 (-0.042,0.139)	0.292	-0.064 (-0.141,0.013)	0.105	0.107 (0.006,0.209)	0.038	3479
Christmas	jan 01-14	All	0.060 (0.004,0.115)	0.035	-0.073 (-0.123,-0.022)	0.005	0.011 (-0.046,0.067)	0.714	6716
Christmas	jan 01-14	Low Intensity	0.082 (-0.006,0.169)	0.067	-0.091 (-0.166,-0.015)	0.018	-0.049 (-0.119,0.022)	0.178	3429
Christmas	jan 01-14	High Intensity	0.020 (-0.069,0.110)	0.654	-0.047 (-0.126,0.032)	0.241	0.095 (-0.003,0.194)	0.058	3287

*This Table gives the estimate of the treatment coefficient in equation (2). An interaction with High Covid-19 Baseline was added to the equation. The outcome is the Inverse Hyperbolic Sine of the Fortnightly Cases, during a period which starts 5 to 7 days after the event (Thanksgiving or Christmas). 95% CI are reported in parentheses. High Baseline is defined as: Cumulative Covid-19 zip cases at baseline above median.

Supplemental Table 3. Analyses of Mobility Outcomes by Party Majority*

			OLS model						Number of days*counties
Campaign	Outcome	Period	High county x Majority Rep	p-value	High county	p-value	Majority Rep	p-value	
Both campaigns	Distance Traveled	from d-3 to d-1	-0.949 (-2.172,0.274)	0.128	-0.240 (-1.211,0.731)	0.628	0.881 (-0.267,2.030)	0.133	4059
Both campaigns	Share Ever Left Home	Thanksgiving (Nov 26)/ Christmas (Dec 24-25)	0.024 (-0.939,0.988)	0.960	0.011 (-0.848,0.869)	0.981	0.009 (-0.606,0.624)	0.977	2017
Thanksgiving	Distance Traveled	from d-3 to d-1	-0.632 (-2.545,1.282)	0.518	-0.422 (-2.067,1.223)	0.615	0.448 (-1.087,1.983)	0.567	2072
Thanksgiving	Share Ever Left Home	Thanksgiving (Nov 26)	0.085 (-1.096,1.265)	0.888	-0.056 (-1.125,1.013)	0.918	-0.143 (-0.920,0.635)	0.719	689
Christmas	Distance Traveled	from d-3 to d-1	-1.472 (-3.208,0.264)	0.097	0.122 (-1.340,1.585)	0.870	1.475 (0.240,2.711)	0.019	1987
Christmas	Share Ever Left Home	Christmas (Dec 24-25)	-0.245 (-1.156,0.666)	0.598	0.287 (-0.513,1.087)	0.482	0.280 (-0.352,0.911)	0.385	1328

*This Table gives the estimates of equation (1) coefficients (here, an interaction with Republican Majority was added to the equation) . Standard errors are clustered at the county level. 95% CI are reported in parentheses. Republican Majority is defined by "share of republican voters > share of democrat voters" in the county.

Supplemental Table 4. Analyses of Covid Outcome by Party Majority*

			OLS model				Number of zip codes
Campaign	Period	County treatment	Treated x Majority Rep	p-value	Treated	p-value	
Both campaigns	dec/jan 01-14	All	-0.001 (-0.052,0.050)	0.975	-0.034 (-0.073,0.005)	0.087	13489
Both campaigns	dec/jan 01-14	Low Intensity	-0.044 (-0.112,0.024)	0.209	-0.003 (-0.051,0.045)	0.901	6723
Both campaigns	dec/jan 01-14	High Intensity	0.001 (-0.071,0.073)	0.979	-0.040 (-0.095,0.015)	0.156	6766
Thanksgiving	dec 01-14	All	-0.046 (-0.111,0.019)	0.164	0.004 (-0.047,0.054)	0.886	6773
Thanksgiving	dec 01-14	Low Intensity	-0.046 (-0.144,0.053)	0.360	0.016 (-0.062,0.094)	0.692	3294
Thanksgiving	dec 01-14	High Intensity	-0.047 (-0.132,0.039)	0.286	-0.008 (-0.073,0.057)	0.817	3479
Christmas	jan 01-14	All	-0.017 (-0.077,0.043)	0.572	-0.031 (-0.076,0.014)	0.175	6716
Christmas	jan 01-14	Low Intensity	-0.063 (-0.143,0.017)	0.123	-0.008 (-0.063,0.047)	0.780	3429
Christmas	jan 01-14	High Intensity	0.032 (-0.059,0.123)	0.491	-0.057 (-0.130,0.015)	0.122	3287

*This Table gives the estimate of the treatment coefficient in equation (2). An interaction with Republican Majority was added to the equation. The outcome is the Inverse Hyperbolic Sine of the Fortnightly Cases, during a period which starts 5 to 7 days after the event (Thanksgiving or Christmas). 95% CI are reported in parentheses. Republican Majority is defined by “share of republican voters > share of democrat voters” in the county.

Supplemental Table 5. Analyses of Mobility Outcomes: Urban vs Rural*

			OLS model						Number of days*counties
Campaign	Outcome	Period	High county x Majority urban	p-value	High county	p-value	Majority urban	p-value	
Both campaigns	Distance Traveled	from d-3 to d-1	0.089 (-1.130,1.309)	0.886	-1.025 (-1.920,-0.130)	0.025	-0.497 (-1.512,0.517)	0.337	4056
Both campaigns	Share Ever Left Home	Thanksgiving (Nov 26)/ Christmas (Dec 24-25)	-0.385 (-1.157,0.386)	0.327	0.203 (-0.343,0.750)	0.466	-0.089 (-0.599,0.421)	0.733	2015
Thanksgiving	Distance Traveled	from d-3 to d-1	0.270 (-1.380,1.919)	0.749	-1.027 (-2.302,0.249)	0.115	-0.502 (-1.769,0.765)	0.438	2072
Thanksgiving	Share Ever Left Home	Thanksgiving (Nov 26)	-0.521 (-1.401,0.359)	0.246	0.233 (-0.404,0.870)	0.474	0.197 (-0.414,0.808)	0.527	689
Christmas	Distance Traveled	from d-3 to d-1	0.074 (-1.473,1.621)	0.925	-1.077 (-2.310,0.156)	0.087	-0.701 (-1.852,0.451)	0.233	1984
Christmas	Share Ever Left Home	Christmas (Dec 24-25)	-0.205 (-0.947,0.538)	0.589	0.184 (-0.385,0.753)	0.526	-0.442 (-0.972,0.087)	0.102	1326

*This Table gives the estimates of equation (1) coefficients (here, an interaction with Urban Majority was added to the equation) .

Standard errors are clustered at the county level. 95% CI are reported in parentheses. Urban Majority is defined by a majority of urban zip codes in the county.

Supplemental Table 6. Analyses of Covid Outcome: Urban vs Rural *

			OLS model				Number of zip codes
Campaign	Period	County treatment	Treated x Majority urban	p-value	Treated	p-value	
Both campaigns	dec/jan 01-14	All	0.037 (-0.016,0.090)	0.176	-0.054 (-0.100,-0.008)	0.021	13489
Both campaigns	dec/jan 01-14	Low Intensity	0.059 (-0.014,0.132)	0.114	-0.063 (-0.127,0.001)	0.053	6723
Both campaigns	dec/jan 01-14	High Intensity	0.020 (-0.053,0.092)	0.597	-0.049 (-0.110,0.012)	0.115	6766
Thanksgiving	dec 01-14	All	0.046 (-0.019,0.111)	0.163	-0.051 (-0.104,0.003)	0.062	6773
Thanksgiving	dec 01-14	Low Intensity	0.051 (-0.044,0.146)	0.294	-0.041 (-0.117,0.036)	0.300	3294
Thanksgiving	dec 01-14	High Intensity	0.043 (-0.045,0.130)	0.339	-0.061 (-0.135,0.013)	0.105	3479
Christmas	jan 01-14	All	0.030 (-0.034,0.093)	0.358	-0.058 (-0.113,-0.004)	0.037	6716
Thanksgiving	dec 01-14	Low Intensity	0.054 (-0.037,0.145)	0.246	-0.079 (-0.160,0.003)	0.059	3429
Thanksgiving	dec 01-14	High Intensity	0.006 (-0.083,0.094)	0.900	-0.039 (-0.112,0.034)	0.297	3287

*This Table gives the estimate of the treatment coefficient in equation (2). An interaction with Urban Majority was added to the equation. The outcome is the Inverse Hyperbolic Sine of the Fortnightly Cases, during a period which starts 5 to 7 days after the event (Thanksgiving or Christmas). 95% CI are reported in parentheses. Urban Majority is defined by a majority of urban zip codes in the county.

Supplemental Table 7. Analyses of Mobility Outcomes by Republican Majority x Urban Majority*

			OLS model								Number of days*counties
Campaign	Outcome	Period	High x Majority urban x Majority rep	p-value	High x Majority rep	p-value	High x Majority urban	p-value	High county	p-value	
Both campaigns	Distance Traveled	from d-3 to d-1	0.378 (-2.464,3.219)	0.794	-1.199 (-3.598,1.200)	0.327	-0.446 (-2.864,1.971)	0.718	0.036 (-2.158,2.231)	0.974	4056
Both campaigns	Share Ever Left Home	Thanksgiving (Nov 26)/ Christmas (Dec 24-25)	-0.932 (-3.192,1.328)	0.419	0.490 (-1.455,2.435)	0.621	0.341 (-1.741,2.424)	0.748	-0.231 (-2.092,1.630)	0.808	2015
Thanksgiving	Distance Traveled	from d-3 to d-1	-0.848 (-5.709,4.014)	0.733	-0.069 (-4.485,4.347)	0.976	0.814 (-3.650,5.277)	0.721	-0.964 (-5.184,3.255)	0.654	2072
Thanksgiving	Share Ever Left Home	Thanksgiving (Nov 26)	-0.382 (-3.251,2.486)	0.794	0.153 (-2.409,2.715)	0.907	-0.242 (-2.937,2.453)	0.860	0.097 (-2.377,2.572)	0.938	689
Christmas	Distance Traveled	from d-3 to d-1	0.999 (-3.534,5.533)	0.666	-2.110 (-6.294,2.074)	0.323	-1.111 (-5.264,3.042)	0.600	0.793 (-3.188,4.773)	0.696	1984
Christmas	Share Ever Left Home	Christmas (Dec 24-25)	-1.962 (-4.245,0.321)	0.092	0.957 (-1.106,3.021)	0.363	1.305 (-0.815,3.426)	0.227	-0.667 (-2.646,1.311)	0.508	1326

*This Table gives the estimates of equation (1) coefficients (here, an interaction with Urban Majority and Republican Majority was added to the equation) . Standard errors are clustered at the county level. 95% CI are reported in parentheses. Urban Majority is defined by a majority of urban zip codes in the county. Republican Majority is defined by "share of republican voters > share of democrat voters" in the county.

Supplemental Table 8. Analyses of Covid Outcome by Republican Majority x Urban Majority*

			OLS model								Number of zip codes
Campaign	Period	County treatment	Treated x Majority urban x Majority rep	p-value	Treated x Majority rep	p-value	Treated x Majority urban	p-value	Treated	p-value	
Both campaigns	dec/jan 01-14	All	-0.129 (-0.278,0.021)	0.092	0.113 (-0.026,0.251)	0.112	0.143 (0.008,0.278)	0.038	-0.153 (-0.283,-0.023)	0.021	13489
Both campaigns	dec/jan 01-14	Low Intensity	-0.003 (-0.215,0.208)	0.975	-0.017 (-0.216,0.182)	0.869	0.053 (-0.139,0.246)	0.587	-0.048 (-0.235,0.139)	0.614	6723
Both campaigns	dec/jan 01-14	High Intensity	-0.135 (-0.348,0.078)	0.215	0.109 (-0.089,0.308)	0.279	0.128 (-0.067,0.323)	0.198	-0.146 (-0.333,0.042)	0.128	6766
Thanksgiving	dec 01-14	All	0.110 (-0.080,0.301)	0.255	-0.110 (-0.286,0.066)	0.220	-0.052 (-0.226,0.122)	0.561	0.047 (-0.120,0.213)	0.583	6773
Thanksgiving	dec 01-14	Low Intensity	0.118 (-0.154,0.389)	0.396	-0.109 (-0.356,0.138)	0.386	-0.048 (-0.295,0.198)	0.700	0.055 (-0.178,0.288)	0.644	3294
Thanksgiving	dec 01-14	High Intensity	0.103 (-0.164,0.369)	0.451	-0.109 (-0.358,0.140)	0.390	-0.052 (-0.297,0.193)	0.677	0.037 (-0.200,0.274)	0.761	3479
Christmas	jan 01-14	All	-0.220 (-0.411,-0.030)	0.023	0.157 (-0.022,0.337)	0.086	0.197 (0.021,0.372)	0.028	-0.197 (-0.367,-0.027)	0.023	6716
Christmas	jan 01-14	Low Intensity	-0.084 (-0.401,0.232)	0.602	0.018 (-0.287,0.324)	0.906	0.099 (-0.198,0.397)	0.513	-0.095 (-0.389,0.198)	0.525	3429
Christmas	jan 01-14	High Intensity	-0.322 (-0.562,-0.082)	0.009	0.263 (0.044,0.482)	0.019	0.260 (0.042,0.478)	0.019	-0.265 (-0.470,-0.061)	0.011	3287

*This Table gives the estimate of the treatment coefficient in equation (2). An interaction with Urban Majority and Republican Majority was added to the equation. The outcome is the Inverse Hyperbolic Sine of the Fortnightly Cases, during a period which starts 5 to 7 days after the event (Thanksgiving or Christmas). 95% CI are reported in parentheses. Urban Majority is defined by a majority of urban zip codes in the county. Republican Majority is defined by "share of republican voters > share of democrat voters" in the county.

Supplemental Table 9. Analyses of Covid Outcome by Excluded counties *

			OLS model				Number of zip codes
Campaign	Period	County treatment	Treated x Excluded	p-value	Treated	p-value	
Thanksgiving	dec 01-14	All	-0.149 (-0.352,0.054)	0.151	-0.022 (-0.054,0.010)	0.181	6773
Thanksgiving	dec 01-14	Low Intensity	-0.269 (-0.554,0.016)	0.065	-0.005 (-0.053,0.043)	0.836	3294
Thanksgiving	dec 01-14	High Intensity	-0.015 (-0.300,0.270)	0.920	-0.038 (-0.082,0.005)	0.084	3479

*This Table gives the estimate of the treatment coefficient in equation (2). An interaction with "County exluded from Christmas sample" was added to the equation. The outcome is the Inverse Hyperbolic Sine of the Fortnightly Cases, during a period which starts 5 after the event. 95% CI are reported in parentheses. The heterogeneity variable is a dummy variable indicating whether the county was excluded from the Christmas campaign sample or not.

Supplemental Table 10. Effect of Intervention on Movement Outcomes, with Double Post Lasso Control Variables*

			Mean (95% CI)		OLS model		Number of days * counties
Campaign	Outcome	Period	High county	Low county	High county coef (95% CI)	p-value	
Both campaigns	Distance Traveled	from d-3 to d-1	-4.384 (-4.973,-3.796)	-3.603 (-4.254,-2.952)	-0.950 (-1.558,-0.342)	0.002	4059
Both campaigns	Share Ever Left Home	Thanksgiving (Nov 26)/ Christmas (Dec 24-25)	72.326 (72.012,72.639)	72.381 (72.092,72.670)	-0.008 (-0.380,0.364)	0.968	2017
Thanksgiving	Distance Traveled	from d-3 to d-1	-6.082 (-6.822,-5.341)	-5.320 (-6.113,-4.527)	-0.731 (-1.528,0.067)	0.073	2072
Thanksgiving	Share Ever Left Home	Thanksgiving (Nov 26)	71.308 (70.885,71.731)	71.468 (71.071,71.866)	0.074 (-0.258,0.406)	0.662	689
Christmas	Distance Traveled	from d-3 to d-1	-2.603 (-3.279,-1.927)	-1.823 (-2.588,-1.057)	-1.004 (-1.764,-0.244)	0.010	1987
Christmas	Share Ever Left Home	Christmas (Dec 24-25)	72.859 (72.507,73.210)	72.852 (72.520,73.185)	0.074 (-0.235,0.384)	0.638	1328

*This Table gives the control and treatment means at the county level and different periods, in addition to the estimate of the treatment coefficient in equation (1). Controls (county covariates and state fixed effects) are selected via Double Post Lasso. Standard errors are clustered at the county level. 95% CI are reported in parentheses.

Supplemental Table 11. Effect of Intervention on Mobility: quantile regression*

			Quantile										
			0.10		0.25		0.5		0.75		0.9		Number of days * counties
Campaign	Outcome	Period	High county (CI 95%)	p-value	High county (CI 95%)	p-value	High county (CI 95%)	p-value	High county (CI 95%)	p-value	High county (CI 95%)	p-value	
Both campaigns	Distance Traveled	from d-3 to d-1	-1.206 (-2.064,-0.347)	0.006	-0.666 (-1.205,-0.128)	0.015	-0.575 (-1.021,-0.130)	0.011	-0.905 (-1.350,-0.461)	0.000	-1.563 (-2.529,-0.598)	0.002	4059
Both campaigns	Share Ever Left Home	Thanksgiving (Nov 26) or Christmas (Dec 24-25)	-0.137 (-0.841,0.566)	0.702	-0.090 (-0.643,0.462)	0.749	-0.172 (-0.752,0.407)	0.560	0.190 (-0.190,0.571)	0.326	0.291 (-0.088,0.670)	0.133	2017
Thanksgiving	Distance Traveled	from d-3 to d-1	-1.223 (-2.202,-0.244)	0.014	-0.836 (-1.366,-0.306)	0.002	-0.441 (-1.100,0.218)	0.190	-0.819 (-1.622,-0.016)	0.046	-1.092 (-2.527,0.342)	0.136	2072
Thanksgiving	Share Ever Left Home	Thanksgiving (Nov 26)	0.304 (-0.862,1.470)	0.609	-0.201 (-0.947,0.544)	0.597	0.103 (-0.482,0.688)	0.731	-0.178 (-0.770,0.415)	0.557	0.235 (-0.466,0.936)	0.512	689
Christmas	Distance Traveled	from d-3 to d-1	-0.970 (-1.985,0.044)	0.061	-0.345 (-0.910,0.221)	0.233	-0.722 (-1.307,-0.137)	0.016	-1.441 (-2.216,-0.666)	0.000	-1.215 (-2.589,0.160)	0.083	1987
Christmas	Share Ever Left Home	Christmas (Dec 24-25)	-0.158 (-0.978,0.662)	0.706	0.170 (-0.325,0.664)	0.501	0.027 (-0.348,0.402)	0.886	0.299 (-0.029,0.626)	0.074	0.319 (-0.083,0.720)	0.120	1328

* This Table gives the quantile treatment effects on mobility at the county level. The coefficients were estimated with the Barrodale and Roberts algorithm (quantreg R package). Standard errors were obtained with the bootstrap method. 95% CI are reported in parentheses.

Supplemental Table 12. Effect of Intervention on Covid-19 Outcome (Asinh(Fortnightly Cases)): quantile regression*

			Quantile										
			0.10		0.25		0.5		0.75		0.9		Number of zip codes
Campaign	Period	County treatment	Treated (CI 95%)	p-value	Treated (CI 95%)	p-value	Treated (CI 95%)	p-value	Treated (CI 95%)	p-value	Treated (CI 95%)	p-value	
Both campaigns	dec/jan 01-14	All	-0.066 (-0.129,-0.003)	0.041	-0.048 (-0.081,-0.015)	0.005	-0.024 (-0.047,-0.001)	0.037	-0.023 (-0.051,0.006)	0.119	-0.010 (-0.048,0.027)	0.587	13489
Both campaigns	dec/jan 01-14	Low Intensity	-0.110 (-0.194,-0.027)	0.010	-0.032 (-0.073,0.009)	0.129	-0.010 (-0.037,0.017)	0.467	-0.003 (-0.036,0.030)	0.864	-0.025 (-0.071,0.021)	0.281	6723
Both campaigns	dec/jan 01-14	High Intensity	-0.054 (-0.135,0.027)	0.192	-0.039 (-0.078,0.001)	0.057	-0.027 (-0.053,-0.001)	0.041	-0.038 (-0.076,-0.001)	0.047	-0.038 (-0.102,0.026)	0.249	6766
Thanksgiving	dec 01-14	All	-0.080 (-0.138,-0.022)	0.007	-0.032 (-0.065,0.001)	0.058	-0.016 (-0.040,0.008)	0.197	-0.011 (-0.037,0.015)	0.404	-0.036 (-0.094,0.023)	0.237	6773
Thanksgiving	dec 01-14	Low Intensity	-0.101 (-0.182,-0.021)	0.013	-0.029 (-0.090,0.031)	0.337	-0.003 (-0.035,0.029)	0.854	-0.004 (-0.039,0.031)	0.813	-0.007 (-0.070,0.057)	0.839	3294
Thanksgiving	dec 01-14	High Intensity	-0.052 (-0.138,0.034)	0.239	-0.033 (-0.084,0.017)	0.190	-0.035 (-0.071,0.001)	0.054	-0.016 (-0.054,0.021)	0.392	-0.062 (-0.151,0.026)	0.169	3479
Christmas	jan 01-14	All	-0.065 (-0.127,-0.003)	0.040	-0.023 (-0.057,0.011)	0.189	-0.016 (-0.040,0.008)	0.197	-0.028 (-0.049,-0.007)	0.008	-0.057 (-0.111,-0.003)	0.040	6716
Christmas	jan 01-14	Low Intensity	-0.099 (-0.218,0.020)	0.104	-0.034 (-0.073,0.005)	0.084	-0.003 (-0.035,0.029)	0.854	-0.010 (-0.037,0.017)	0.462	-0.030 (-0.104,0.044)	0.426	3429
Christmas	jan 01-14	High Intensity	-0.029 (-0.142,0.083)	0.609	-0.019 (-0.076,0.039)	0.528	-0.035 (-0.071,0.001)	0.054	-0.038 (-0.075,-0.001)	0.042	-0.081 (-0.159,-0.004)	0.039	3287

*This Table gives the quantile treatment effects on Covid-19 cases at the zip level. The outcome is Inverse Hyperbolic Sine of the Fortnightly Cases, during a period which starts 5 to 7 days after the event (Thanksgiving or Christmas). 95% CI are reported in parentheses. The coefficients were estimated with the Barrodale and Roberts algorithm (quantreg R package). Standard errors were obtained with the bootstrap method. 95% CI are reported in parentheses.

Supplemental Table 13. Effect of Intervention on Covid-19 Outcome (Log(Fortnightly Cases +1)): quantile regression*

			Quantile										
			0.10		0.25		0.5		0.75		0.9		Number of zip codes
Campaign	Period	County treatment	Treated (CI 95%)	p-value	Treated (CI 95%)	p-value	Treated (CI 95%)	p-value	Treated (CI 95%)	p-value	Treated (CI 95%)	p-value	
Both campaigns	dec/jan 01-14	All	-0.058 (-0.105,-0.010)	0.017	-0.044 (-0.081,-0.007)	0.019	-0.020 (-0.043,0.004)	0.099	-0.024 (-0.051,0.003)	0.087	0.000 (-0.045,0.045)	0.993	13489
Both campaigns	dec/jan 01-14	Low Intensity	-0.091 (-0.167,-0.016)	0.017	-0.032 (-0.069,0.004)	0.084	-0.002 (-0.029,0.025)	0.867	-0.005 (-0.040,0.031)	0.802	-0.024 (-0.065,0.016)	0.242	6723
Both campaigns	dec/jan 01-14	High Intensity	-0.051 (-0.138,0.036)	0.249	-0.039 (-0.087,0.009)	0.110	-0.025 (-0.051,0.001)	0.056	-0.032 (-0.070,0.007)	0.111	-0.042 (-0.109,0.026)	0.227	6766
Thanksgiving	dec 01-14	All	-0.060 (-0.110,-0.009)	0.022	-0.021 (-0.062,0.020)	0.315	-0.004 (-0.026,0.018)	0.703	-0.018 (-0.048,0.013)	0.258	-0.032 (-0.078,0.013)	0.166	6773
Thanksgiving	dec 01-14	Low Intensity	-0.068 (-0.146,0.009)	0.085	-0.025 (-0.091,0.041)	0.461	0.010 (-0.028,0.047)	0.609	-0.006 (-0.049,0.038)	0.802	0.001 (-0.054,0.056)	0.968	3294
Thanksgiving	dec 01-14	High Intensity	-0.047 (-0.110,0.015)	0.138	-0.025 (-0.072,0.023)	0.306	-0.015 (-0.045,0.015)	0.334	-0.026 (-0.065,0.012)	0.185	-0.062 (-0.121,-0.003)	0.040	3479
Christmas	jan 01-14	All	-0.057 (-0.117,0.004)	0.066	-0.024 (-0.058,0.011)	0.174	-0.021 (-0.042,0.000)	0.051	-0.027 (-0.048,-0.005)	0.014	-0.036 (-0.086,0.015)	0.168	6716
Christmas	jan 01-14	Low Intensity	-0.087 (-0.201,0.028)	0.138	-0.033 (-0.072,0.006)	0.100	-0.006 (-0.040,0.028)	0.721	-0.017 (-0.042,0.009)	0.207	-0.022 (-0.086,0.041)	0.491	3429
Christmas	jan 01-14	High Intensity	-0.044 (-0.129,0.040)	0.303	-0.010 (-0.070,0.051)	0.751	-0.033 (-0.066,0.000)	0.051	-0.045 (-0.082,-0.007)	0.021	-0.058 (-0.131,0.015)	0.118	3287

*This Table gives the quantile treatment effects on Covid-19 cases at the zip level. The outcome is log(Fortnightly Cases +1), during a period which starts 5 to 7 days after the event (Thanksgiving or Christmas). 95% CI are reported in parentheses. The coefficients were estimated with the Barrodale and Roberts algorithm (quantreg R package). Standard errors were obtained with the bootstrap method. 95% CI are reported in parentheses.

Supplemental Table 14. Effect of Intervention on Covid-19 Outcome (both campaigns), robustness to function form

				Mean (CI 95%)		OLS model		Number of zip codes
Specification	Outcome	Period	County treatment	Treatment	Control	Treatment (CI 95%)	p-value	
1	Log(Fortnightly Cases)	dec/jan 01-14	All	3.718 (3.672,3.764)	3.745 (3.700,3.790)	-0.033 (-0.060,-0.007)	0.013	13269
1	Log(Fortnightly Cases)	dec/jan 01-14	Low Intensity	3.733 (3.649,3.816)	3.738 (3.687,3.788)	-0.036 (-0.070,-0.001)	0.042	6603
1	Log(Fortnightly Cases)	dec/jan 01-14	High Intensity	3.713 (3.663,3.764)	3.767 (3.688,3.847)	-0.034 (-0.069,0.000)	0.051	6666
2	Log(Fortnightly Cases)	dec/jan 01-14	All	3.649 (3.601,3.697)	3.670 (3.623,3.717)	-0.036 (-0.064,-0.008)	0.011	13489
2	Log(Fortnightly Cases)	dec/jan 01-14	Low Intensity	3.657 (3.570,3.744)	3.657 (3.604,3.711)	-0.034 (-0.070,0.002)	0.066	6723
2	Log(Fortnightly Cases)	dec/jan 01-14	High Intensity	3.646 (3.593,3.699)	3.707 (3.624,3.790)	-0.040 (-0.076,-0.003)	0.033	6766
3	Log(Fortnightly Cases+1)	dec/jan 01-14	All	3.732 (3.687,3.777)	3.750 (3.706,3.794)	-0.030 (-0.054,-0.005)	0.020	13489
3	Log(Fortnightly Cases+1)	dec/jan 01-14	Low Intensity	3.745 (3.664,3.826)	3.739 (3.689,3.788)	-0.025 (-0.057,0.007)	0.128	6723
3	Log(Fortnightly Cases+1)	dec/jan 01-14	High Intensity	3.728 (3.679,3.777)	3.784 (3.707,3.861)	-0.035 (-0.068,-0.003)	0.033	6766

*This Table gives the control and treatment means at the zip level, in addition to the estimate of the treatment coefficient in equation (2). The outcome is a function of the Fortnightly Cases, during a period which starts 5 to 7 days after the event (Thanksgiving or Christmas). 95% CI are reported in parentheses. Standard errors are clustered at the zip level. Specification 1 = "Fortnightly cases zeros are omitted." Specification 2 = "Fortnightly cases zeros are replaced with min(positive Fortnightly cases)/2". Specification 3 = "Adding 1."

Supplemental Table 15. Effect of Intervention on Covid-19 Outcome (Thanksgiving campaign), robustness to functional form

				Mean (CI 95%)		OLS model		Number of zip codes
Specification	Outcome	Period	County treatment	Treatment	Control	Treatment (CI 95%)	p-value	
1	Log(Fortnightly Cases)	dec/jan 01-14	All	3.700 (3.646,3.753)	3.660 (3.607,3.713)	-0.022 (-0.053,0.010)	0.172	6672
1	Log(Fortnightly Cases)	dec/jan 01-14	Low Intensity	3.651 (3.540,3.762)	3.628 (3.567,3.690)	-0.025 (-0.072,0.021)	0.288	3239
1	Log(Fortnightly Cases)	dec/jan 01-14	High Intensity	3.715 (3.654,3.776)	3.748 (3.644,3.853)	-0.019 (-0.061,0.024)	0.383	3433
2	Log(Fortnightly Cases)	dec/jan 01-14	All	3.632 (3.576,3.687)	3.597 (3.542,3.652)	-0.028 (-0.061,0.004)	0.089	6773
2	Log(Fortnightly Cases)	dec/jan 01-14	Low Intensity	3.582 (3.466,3.698)	3.555 (3.490,3.619)	-0.017 (-0.066,0.032)	0.495	3294
2	Log(Fortnightly Cases)	dec/jan 01-14	High Intensity	3.647 (3.584,3.711)	3.718 (3.612,3.824)	-0.039 (-0.083,0.005)	0.079	3479
3	Log(Fortnightly Cases+1)	dec/jan 01-14	All	3.714 (3.663,3.766)	3.679 (3.627,3.730)	-0.021 (-0.050,0.007)	0.145	6773
3	Log(Fortnightly Cases+1)	dec/jan 01-14	Low Intensity	3.670 (3.563,3.778)	3.639 (3.580,3.699)	-0.010 (-0.053,0.032)	0.635	3294
3	Log(Fortnightly Cases+1)	dec/jan 01-14	High Intensity	3.728 (3.669,3.787)	3.791 (3.691,3.890)	-0.032 (-0.071,0.007)	0.108	3479

*This Table gives the control and treatment means at the zip level, in addition to the estimate of the treatment coefficient in equation (2). The outcome is a function of the Fortnightly Cases, during a period which starts 5 to 7 days after the event (Thanksgiving). 95% CI are reported in parentheses. Standard errors are clustered at the zip level. Specification 1 = "Fortnightly cases zeros are omitted." Specification 2 = "Fortnightly cases zeros are replaced with min(positive Fortnightly cases)/2". Specification 3 = "Adding 1."

Supplemental Table 16. Effect of Intervention on Covid-19 Outcome (Christmas campaign), robustness to functional form

				Mean (CI 95%)		OLS model		Number of zip codes
Specification	Outcome	Period	County treatment	Treatment	Control	Treatment (CI 95%)	p-value	
1	Log(Fortnightly Cases)	dec/jan 01-14	All	3.737 (3.681,3.793)	3.830 (3.775,3.884)	-0.049 (-0.078,-0.020)	0.001	6597
1	Log(Fortnightly Cases)	dec/jan 01-14	Low Intensity	3.810 (3.696,3.924)	3.844 (3.781,3.906)	-0.050 (-0.090,-0.010)	0.015	3364
1	Log(Fortnightly Cases)	dec/jan 01-14	High Intensity	3.711 (3.647,3.775)	3.787 (3.678,3.896)	-0.049 (-0.090,-0.007)	0.021	3233
2	Log(Fortnightly Cases)	dec/jan 01-14	All	3.666 (3.608,3.724)	3.742 (3.684,3.799)	-0.044 (-0.075,-0.013)	0.006	6716
2	Log(Fortnightly Cases)	dec/jan 01-14	Low Intensity	3.727 (3.608,3.846)	3.757 (3.691,3.823)	-0.051 (-0.094,-0.008)	0.021	3429
2	Log(Fortnightly Cases)	dec/jan 01-14	High Intensity	3.645 (3.578,3.711)	3.695 (3.580,3.811)	-0.037 (-0.082,0.008)	0.109	3287
3	Log(Fortnightly Cases+1)	dec/jan 01-14	All	3.750 (3.696,3.804)	3.821 (3.767,3.874)	-0.038 (-0.064,-0.011)	0.006	6716
3	Log(Fortnightly Cases+1)	dec/jan 01-14	Low Intensity	3.815 (3.704,3.925)	3.835 (3.773,3.897)	-0.039 (-0.076,-0.002)	0.041	3429
3	Log(Fortnightly Cases+1)	dec/jan 01-14	High Intensity	3.728 (3.666,3.789)	3.777 (3.670,3.884)	-0.036 (-0.075,0.002)	0.065	3287

*This Table gives the control and treatment means at the zip level, in addition to the estimate of the treatment coefficient in equation (2). The outcome is a function of the Fortnightly Cases, during a period which starts 5 to 7 days after the event (Christmas). 95% CI are reported in parentheses. Standard errors are clustered at the zip level. Specification 1 = "Fortnightly cases zeros are omitted." Specification 2 = "Fortnightly cases zeros are replaced with min(positive Fortnightly cases)/2". Specification 3 = "Adding 1."
