# Supplementary material for: Effects of a large-scale social media advertising campaign on holiday travel and COVID-19 infections: a cluster randomized controlled trial
Source: Nat Med. 2021 Aug 19;27(9):1622–8. doi: 10.1038/s41591-021-01487-3 (PMC8440209; doi:10.1038/s41591-021-01487-3)
Supplement: Supplementary file 2 — Reporting Summary [file 41591_2021_1487_MOESM2_ESM.pdf]

nature portfolio

Corresponding author(s): Esther Duflo

Last updated by author(s): Jul 28, 2021

Reporting Summary

Nature Portfolio wishes to improve the reproducibility of the work that we publish. This form provides structure for consistency and transparency in reporting. For further information on Nature Portfolio policies, see our Editorial Policies and the Editorial Policy Checklist.

Statistics

For all statistical analyses, confirm that the following items are present in the figure legend, table legend, main text, or Methods section.

n/a	Confirmed	
☐	☒	The exact sample size (n) for each experimental group/condition, given as a discrete number and unit of measurement
☐	☒	A statement on whether measurements were taken from distinct samples or whether the same sample was measured repeatedly
☐	☒	The statistical test(s) used AND whether they are one- or two-sided<br>*Only common tests should be described solely by name; describe more complex techniques in the Methods section.*
☐	☒	A description of all covariates tested
☐	☒	A description of any assumptions or corrections, such as tests of normality and adjustment for multiple comparisons
☐	☒	A full description of the statistical parameters including central tendency (e.g. means) or other basic estimates (e.g. regression coefficient) AND variation (e.g. standard deviation) or associated estimates of uncertainty (e.g. confidence intervals)
☐	☒	For null hypothesis testing, the test statistic (e.g. F, t, r) with confidence intervals, effect sizes, degrees of freedom and P value noted<br>*Give P values as exact values whenever suitable.*
☒	☐	For Bayesian analysis, information on the choice of priors and Markov chain Monte Carlo settings
☐	☒	For hierarchical and complex designs, identification of the appropriate level for tests and full reporting of outcomes
☒	☐	Estimates of effect sizes (e.g. Cohen's d, Pearson's r), indicating how they were calculated

Our web collection on statistics for biologists contains articles on many of the points above.

Software and code

Policy information about availability of computer code

Data collection	Computer code was not used to collect data.
Data analysis	Analyses were performed using R, version 4.0.3, including the following packages (versions): stats (4.0.3), tidyverse (1.3.0), estimatr (0.28.0), readr (1.4.0), dplyr (1.0.5), lubridate (1.7.10), hdm (0.3.1), car (3.0.10), MASS (7.3.53), sandwich (3.0.0), foreign (0.8.80), readstata13 (0.9.2), readxl (1.3.1), quantreg (5.75). The data and all the statistical codes are available at https://doi.org/10.7910/DVN/4EK4KX.

For manuscripts utilizing custom algorithms or software that are central to the research but not yet described in published literature, software must be made available to editors and reviewers. We strongly encourage code deposition in a community repository (e.g. GitHub). See the Nature Portfolio guidelines for submitting code & software for further information.

Data

Policy information about availability of data

All manuscripts must include a data availability statement. This statement should provide the following information, where applicable:

- Accession codes, unique identifiers, or web links for publicly available datasets
- A description of any restrictions on data availability
- For clinical datasets or third party data, please ensure that the statement adheres to our policy

All Facebook data used in the analysis is publicly available to anyone at https://dataforgood.fb.com/docs/covid19/ and no restricted data was used to generate the primary outcomes.
The county-level state COVID-19 case data were retrieved from the following state websites:
AZ: https://www.azdhs.gov/covid19/data/index.php
AR: https://achi.net/covid19/

FL: https://experience.arcgis.com/experience/96dd742462124fa0b38ddedb9b25e429
IL: https://www.dph.illinois.gov/covid19/covid19-statistics
IN: https://hub.mph.in.gov/dataset?q=COVID
ME: https://www.maine.gov/dhhs/mecdc/infectious-disease/epi/airborne/coronavirus/data.shtml
MD: https://coronavirus.maryland.gov/datasets/mdcovid19-master-zip-code-cases/data
MN: https://www.health.state.mn.us/diseases/coronavirus/stats/index.html
NC: https://covid19.ncdhhs.gov/dashboard
OK: https://looker-dashboards.ok.gov/embed/dashboards/80
OR: https://govstatus.egov.com/OR-OHA-COVID-19
RI: https://ri-department-of-health-covid-19-data-rihealth.hub.arcgis.com/
VA: https://www.vdh.virginia.gov/coronavirus/covid-19-data-insights/
All data are shared in a public registry (Harvard MIT data archive). The data are freely accessible at https://doi.org/10.7910/DVN/4EK4KX.

Field-specific reporting

Please select the one below that is the best fit for your research. If you are not sure, read the appropriate sections before making your selection.

☐ Life sciences ☒ Behavioural & social sciences ☐ Ecological, evolutionary & environmental sciences

For a reference copy of the document with all sections, see nature.com/documents/nr-reporting-summary-flat.pdf

Behavioural & social sciences study design

All studies must disclose on these points even when the disclosure is negative.

Study description	The study reports results from a randomized controlled trial in the US which sent clinician-recorded video messages to 34 million Facebook users encouraging viewers to stay at home during COVID-19. The data is quantitative.
Research sample	Our research sample consisted of movement range data from 820 counties covering 13 states, as well as COVID-19 case counts from 6,998 zip codes in the aforementioned 820 counties. The sample was chosen to include the states that published zip code-level COVID-19 case counts. The Facebook ad campaigns aimed to reach as many Facebook users as possible in treated zip codes. Approximately 70% of US adults are Facebook users.
Sampling strategy	We selected 13 states where weekly COVID-19 case counts were available at the zip code level prior to our study's launch, and selected counties within these states where these data were available.
Data collection	The study did not involve any new primary data collection. All outcomes come from publicly-available sources. The mobility data is from Facebook's Movement Range Data, which is published by Facebook Data for Good. The COVID-19 case count data was downloaded from the states' websites.
Timing	Data were collected from November 2020 to January 2021.
Data exclusions	Messages were not randomized to zip codes that had missing COVID-19 case data or could not be matched to county-level census data. Prior to Christmas, 60 counties in the top tercile of 2020 Donald Trump vote share were removed due to concerns about unintended adverse effects of messages given growing political polarization in December 2020-January 2021.
Non-participation	No participants dropped out or declined participation because we did not recruit individuals or use individual-level data.
Randomization	Randomization of messages to Facebook users was conducted as follows. First, counties were randomized into high- and low-intensity groups. Then, zip codes within counties were randomly assigned to treatment or control such that 75% of zip codes in high-intensity counties received treatment, and 25% of zip codes in low-intensity counties received treatment. In selected zip codes, as many users were reached as possible.

Reporting for specific materials, systems and methods

We require information from authors about some types of materials, experimental systems and methods used in many studies. Here, indicate whether each material, system or method listed is relevant to your study. If you are not sure if a list item applies to your research, read the appropriate section before selecting a response.

Materials & experimental systems

n/a	Involved in the study
☒	☐ Antibodies
☒	☐ Eukaryotic cell lines
☒	☐ Palaeontology and archaeology
☒	☐ Animals and other organisms
☒	☐ Human research participants
☐	☒ Clinical data
☒	☐ Dual use research of concern

Methods

n/a	Involved in the study
☒	☐ ChIP-seq
☒	☐ Flow cytometry
☒	☐ MRI-based neuroimaging

Clinical data

Policy information about clinical studies

All manuscripts should comply with the ICMJE guidelines for publication of clinical research and a completed CONSORT checklist must be included with all submissions.

Clinical trial registration

The study was registered on clinicaltrials.gov (NCT04644328) and the AER registry for randomized social experiments (AEARCTR-0006821).

Study protocol

The registration documents contain all of the study protocols. The research team had no contact with any of the study subjects.

Data collection

The research team did not collect any data directly from participants. All clinical data was downloaded from US state websites.

Outcomes

One of our primary outcome is the number of new COVID-19 cases detected in each zip code during fortnight that starts five days after each holiday: given the incubation period of five days, this is the one two-week period where we should see an impact. We use an inverse hyperbolic sine transformation, which is appropriate when the data is approximately lognormal for higher values, but a small number of observations have zero cases. We chose to transform the fortnightly cases with this function, because it has the property of being equivalent to x close to 0 and equivalent to ln(x) when x is large.

We pre-registered that we would analyze COVID-19 infections as one of our primary outcomes. However, we had not specified a functional form for the number of COVID-19 cases.
